# *Notes From the Field:* Xylazine-Related Deaths — Cook County, Illinois, 2017–2021

**DOI:** 10.15585/mmwr.mm7113a3

**Published:** 2022-04-01

**Authors:** Neeraj Chhabra, Mojde Mir, Miao Jenny Hua, Sarah Berg, Juleigh Nowinski-Konchak, Steve Aks, Ponni Arunkumar, Keiki Hinami

**Affiliations:** ^1^Division of Medical Toxicology, Department of Emergency Medicine, Cook County Health, Chicago, Illinois; ^2^Toxikon Consortium, Chicago, Illinois; ^3^Cook County Medical Examiner’s Office, Chicago, Illinois; ^4^Department of Preventive Medicine, Cook County Health, Chicago, Illinois; ^5^Feinberg School of Medicine, Northwestern University, Chicago Illinois; ^6^Center for Health Equity and Innovation, Cook County Health, Chicago, Illinois.

Xylazine, an alpha-2 receptor agonist, is used in veterinary medicine as a sedative and muscle relaxant; it is not approved for use in humans. However, reports of adulteration of illicit opioids with xylazine have been increasing in the United States (*1*–*3*). In humans, xylazine can cause respiratory depression, bradycardia, and hypotension (*4*). Typical doses of naloxone are not expected to reverse the effects of xylazine; therefore, persons who use xylazine-adulterated opioids are at high-risk for fatal overdose. Although some regions of the United States have reported increases in xylazine-involved deaths, xylazine was involved in <2% of overdose deaths nationally in 2019 (*2*,*5*). Most xylazine-involved deaths are associated with fentanyls, including fentanyl analogs (*1*,*5*). Cook County, Illinois, is the second largest county in the United States and has a high incidence of opioid-related deaths involving fentanyl (*6*). To determine temporal trends in xylazine-involved deaths in Cook County, the Cook County Medical Examiner’s Office and Cook County Health analyzed suspected substance-related deaths from January 2017 to October 2021 for the presence of xylazine and co-occurring substances.

A xylazine-associated death was defined as a positive postmortem xylazine serum toxicology test result in an unintentional, undetermined, or pending intent substance-related death during January 2017–October 2021. Routine postmortem tests were conducted for other substances including fentanyl, fentanyl analogs, cocaine, and naloxone. Xylazine testing is standard in Cook County for suspected drug overdose deaths. This activity was reviewed by CDC and was conducted consistent with applicable federal law and CDC policy.*

A total of 236 xylazine-associated deaths were reported during the study period. Xylazine-associated deaths increased throughout the study period; incidence peaked during July 2021 (Figure). The percentage of fentanyl-associated deaths involving xylazine also increased throughout the study period, rising to a peak of 11.4% of fentanyl-related deaths assessed by the Cook County Medical Examiner’s Office during October 2021. Fentanyl or fentanyl analogs were detected on forensic testing in most xylazine-involved deaths (99.2%). Other common co-occurring substances included diphenhydramine (79.7%), cocaine (41.1%), and quinine (37.3%). Naloxone was detected in 32.2% of xylazine-associated deaths.

**FIGURE:**
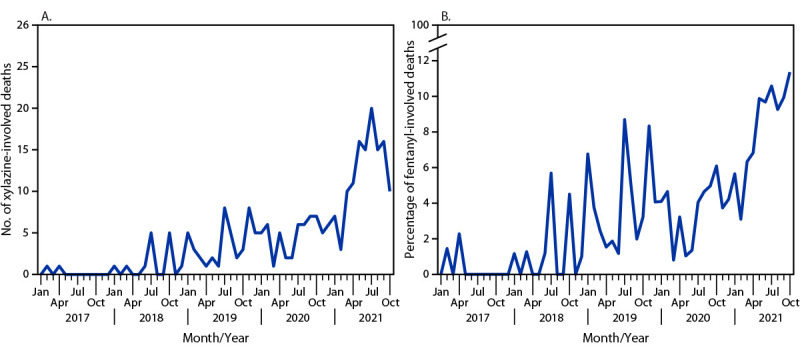
Number of xylazine-involved deaths (A) and percentage of fentanyl-involved deaths with detectable xylazine (B), by month — Cook County, Illinois, 2017–2021

These findings highlight a concerning trend in xylazine-involved deaths in Cook County, Illinois. Increased monitoring and public education within Cook County are warranted along with expanded surveillance in other jurisdictions, particularly those in which fentanyl use is highly prevalent. These findings can be helpful in guiding overdose prevention and response efforts because naloxone has not been shown to reverse the effects of xylazine. Although a specific antidote is not available for xylazine, naloxone should still be administered in suspected cases of potentially fatal overdose because most cases co-occur with opioids. Cardiovascular and respiratory support are critical to the management of serious xylazine toxicity; health care providers should be made aware that cases of suspected fentanyl overdose that are refractory to naloxone administration might involve xylazine toxicity. Designation of xylazine as a controlled substance has occurred in some states and would be an important policy to be considered more broadly.^†^ In addition, expanded postmortem testing for xylazine and co-occurring substances across jurisdictions could better define the role of xylazine in opioid-related deaths.

## Footnotes

* 45 C.F.R. part 46.102(l)(2), 21 C.F.R. part 56; 42 U.S.C. Sect. 241(d); 5 U.S.C. Sect. 552a; 44 U.S.C. Sect. 3501 et seq.

†
https://www.nysenate.gov/newsroom/press-releases/terrence-murphy/slaying-monster-senate-passes-murphys-bill-designating
