# *Erratum*: Vol. 71, No. 13

**DOI:** 10.15585/mmwr.mm7118a5

**Published:** 2022-05-06

In the report “Notes from the Field: Xylazine-Related Deaths — Cook County, Illinois, 2017–2021” on page 503, the second paragraph of the second column should have read, “A total of **210** xylazine-associated deaths were reported during the study period. Xylazine-associated deaths increased throughout the study period; incidence peaked during **October** 2021 (Figure). The percentage of fentanyl-associated deaths involving xylazine also increased throughout the study period, rising to a peak of **12.2%** of fentanyl-related deaths assessed by the Cook County Medical Examiner’s Office during October 2021. Fentanyl or fentanyl analogs were detected on forensic testing in most xylazine-involved deaths **(99.1%)**. Other common co-occurring substances included diphenhydramine **(78.1%)**, cocaine **(41.9%)**, and quinine **(33.8%)**. Naloxone was detected in **33.3%** of xylazine-associated deaths.”

**FIGURE:**
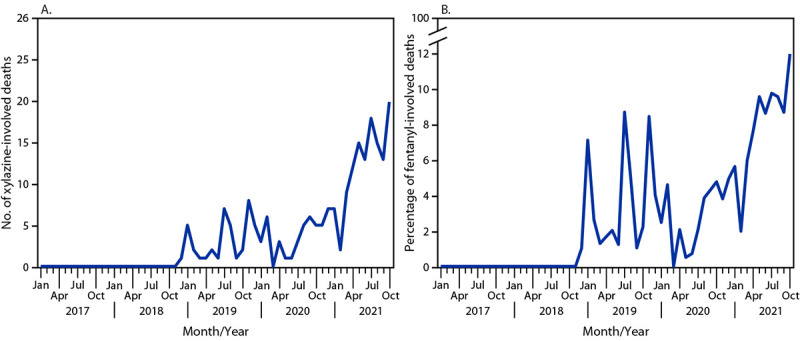
Number of xylazine-involved deaths (A) and percentage of fentanyl-involved deaths with detectable xylazine (B), by month — Cook County, Illinois, 2017–2021

The figure on page 503 was updated accordingly.

